# Immunological and clinical immunotherapy implications of *NLRP3* mutations in melanoma

**DOI:** 10.18632/aging.203678

**Published:** 2021-11-08

**Authors:** Qinghua Wang, Juncheng Lyu, Wenjing Zhang, Fuyan Shi, Yanfeng Ren, Qian Mao, Yujie Liu, Yuting Li, Suzhen Wang

**Affiliations:** 1 Department of Health Statistics, Key Laboratory of Medicine and Health of Shandong Province, School of Public Health, Weifang Medical University, Weifang 261053, Shandong, China; 2 Tianjin Cancer Institute, National Clinical Research Center for Cancer, Key Laboratory of Cancer Prevention and Therapy of Tianjin, Tianjin Medical University Cancer Institute and Hospital, Tianjin 300060, Tianjin, China

**Keywords:** melanoma, immunotherapy, *NLRP3* mutations, predictive indicator, clinical practice

## Abstract

Recent studies have demonstrated the role of Nod-like receptor protein 3 (NLRP3) inflammasome in promoting melanoma progression. Immune checkpoint inhibitors (ICI) treatment dramatically extended the survival outcomes for advanced melanoma patients. Nevertheless, immunologic and immunotherapy implications of *NLRP3* mutations in melanoma were obscure. Herein, we utilized publicly genomic data of 750 melanoma patients to explore the association of *NLRP3* mutations with immunologic and genomic features. In addition, we curated 336 advanced/metastatic melanoma patients treated with ICI agents from 6 published studies to analyze the response rate and survival outcome in relation to *NLRP3* mutations. We observed that patients with *NLRP3* mutations had a significantly higher tumor mutation burden (*P* < 0.001) and neoantigen burden (*P* < 0.001). Moreover, significantly lower tumor heterogeneity (*P* = 0.048) and purity (*P* = 0.022) were also observed in this mutated subgroup. Elevated infiltration of immune-response cells, decreased enrichment of immune-suppressive cells, and immune response-related circuits were markedly enriched in patients with *NLRP3* mutations. In the pooled ICI-treated cohort, *NLRP3* mutations were linked with the higher response rate (*P* = 0.031) and preferable survival outcome (*P* = 0.006). *NLRP3* mutations were identified to associate with the elevated mutational burden, favorable immune infiltration, and preferable ICI efficacy. Findings derived from our study suggest that *NLRP3* mutations may serve as a potential biomarker for evaluating melanoma immunotherapy response.

## INTRODUCTION

Due to the evidently better variability in immunogenicity during tumor progression, melanoma is broadly considered as an immunogenic malignancy [1], which serves as the model system for evaluating the effectiveness of invented immunotherapies [2]. Therefore, therapies for melanoma have been recently changed owing to the emergence of immune checkpoint inhibitors (ICI) including anti-CTLA-4 and anti-PD-1 agents [3]. ICI therapies have observably extended the survival time for advanced melanoma patients [4, 5]. Nevertheless, the remarkable efficacy was only observed in a fraction of patients, most were not benefitted.

Recent studies demonstrated that ICI therapies were influenced by a combination of predictive biomarkers related to genomics, immune checkpoints expression, characteristics of the microenvironment, and gut microbiome [6]. Tumor mutation burden (TMB) and neoantigen burden (NB) emerged as promising markers for evaluating ICI efficacy and previous findings have demonstrated their positive association with the immunotherapy response rate and prognosis via numerous clinical trials [7–9]. However, a few studies concluded controversial results, that is high TMB sometimes could not accurately predict ICI response [8]. Immune checkpoints, such as programmed cell death ligand 1 (PD-L1) expression is another widely used biomarker associated with ICI therapies efficacy. Similarly, it may be out of work in some trials [10]. In view of the current situation, novel and more effective indicators were needed to distinguish subpopulations that are likely to be sensitive to ICI treatment.

Nod-like receptor protein 3 (NLRP3) inflammasome was a three-domain complex associated with inflammation regulation, immune response, and cell apoptosis [11]. Functions of NLRP3 inflammasome in cancer progression remained inconsistent owing to the controversial results reported [12]. For instance, the protective effects of this inflammasome were observed in colon cancer [13, 14]. Conversely, it exhibited a promotion role in cancers of gastric [15], liver [16], head and neck [17], lung [18], prostate [19], glioblastoma [20], and melanoma [21].

Recent studies demonstrated that NLRP3 inflammasome upregulation may inhibit the inflammatory responses in melanoma. Consistently, a mice model with NLRP3 deficiency showed the protection roles against cancer progression [22, 23]. The progression of cancer cells could be suppressed by reduced NLRP3 inflammasome and IL-1β expression [24]. It has been shown that NLRP3 downregulation and reduced IL-1β secretion decreased metastatic melanoma by thymoquinone therapy in a mouse model [21]. Evaluation of the roles of NLRP3 inflammasome in the immune response by employing vaccination against the melanoma cells demonstrated that mice with NLRP3 vaccination deficiency who received a subcutaneous injection of poorly immunogenic melanoma cells leading to a 4-fold promotion in survival times as compared to the control mice [25]. NLRP3 plays vital roles in melanoma tumorigenesis, progression, and immune response, however, its alterations association with ICI efficacy remains unclear.

Herein, we analyzed whether *NLRP3* mutations were correlated with immunological and genomic features with publicly available data in melanoma. Further, the association of *NLRP3* mutations with ICI efficacy was estimated with an aggregated ICI-treated cohort. Novel findings would provide implications for tailoring clinical trials and immunotherapeutic strategies for melanoma.

## RESULTS

### *NLRP3* mutations in melanoma

**Figure 1:**
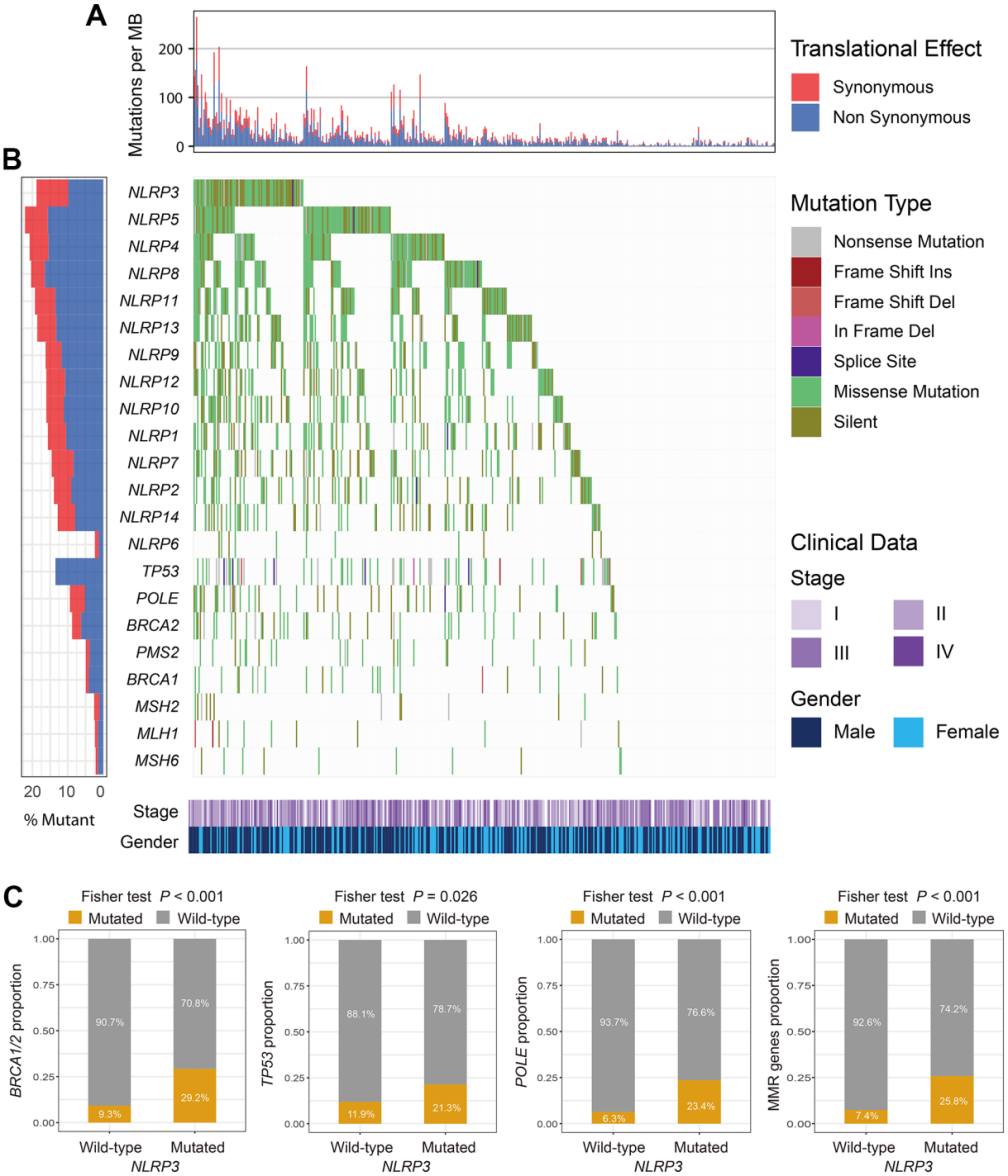
**The mutational patterns of NLR family members and genome maintenance genes.** (**A**) TMB stratified by synonymous and non-synonymous mutations for each patient. (**B**) Waterfall plot for NLR family members and genome maintenance genes. (**C**) Association of *NLRP3* mutations with *BRCA1/2*, *TP53*, *POLE*, and MMR genes mutations.

In the TCGA cohort, 89 (19.1%) of 467 melanoma patients harbored *NLRP3* mutations. *NLRP3* is one frequently mutated gene and we found that patients with *NLRP3* mutations had higher TML as compared with others (Figure 1A). Of 89 *NLRP3* mutated patients, 53 (59.5%) also had mutations of genes related to genomic maintenance including *TP53*, *BRCA1/2*, *POLE,* and MMR genes (Figure 1B). Further analyses revealed that *NLRP3* mutated patients harbored significantly higher mutation rates of above genome repair genes than *NLRP3* wild-type patients (Fisher exact test, all *P* < 0.05; Figure 1C). Mutational patterns of *NLRP3*, its family members, and genomic maintenance genes were exhibited in Figure 1B.

### *NLRP3* mutations were correlated with high TMB, NB, and favorable genomic features

**Figure 2:**
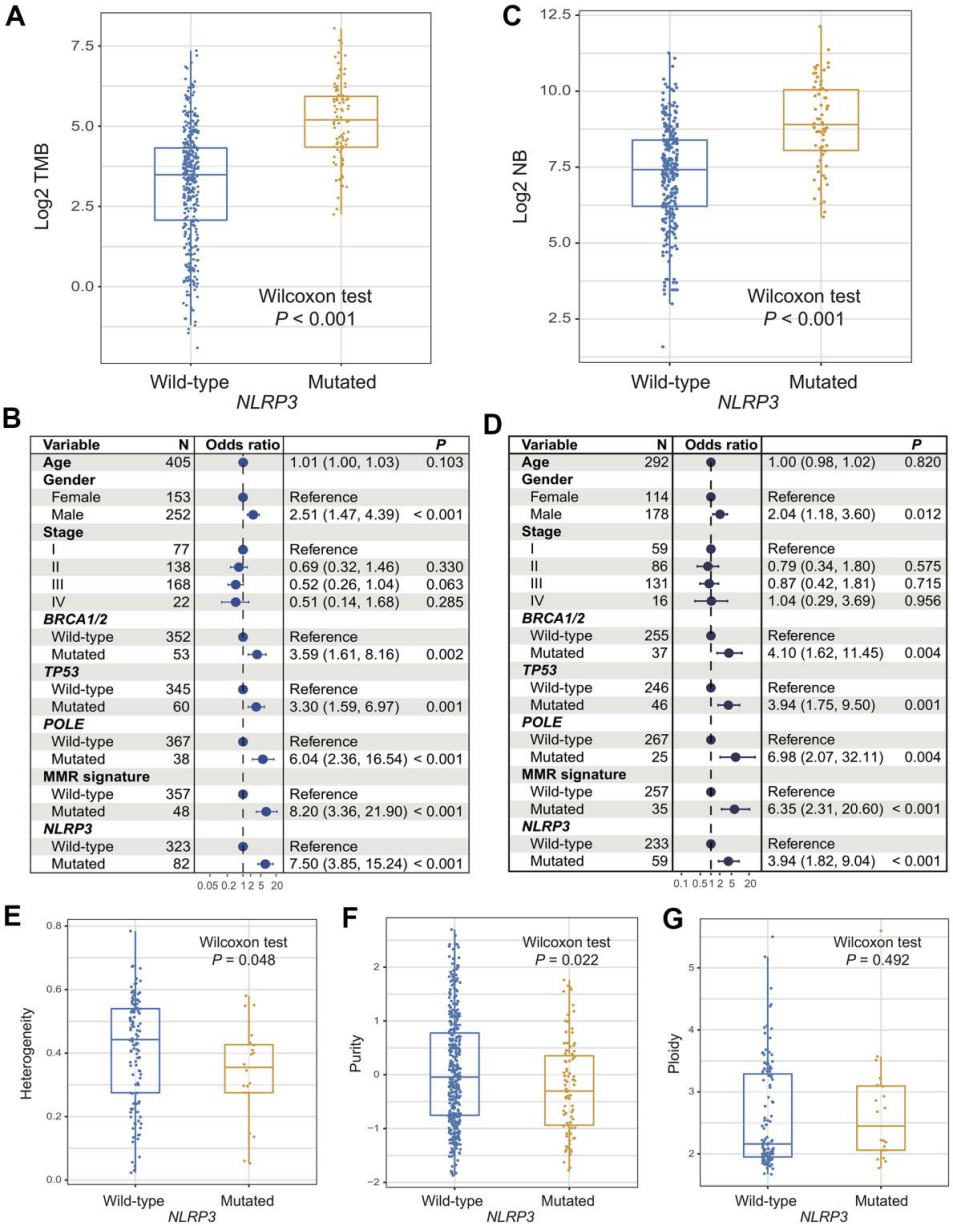
**Association of *NLRP3* mutations with TMB, NB, and genomic features.** (**A**, **B**) *NLRP3* mutations versus TMB with univariate analysis and multivariate regression model. (**C**, **D**) *NLRP3* mutations versus NB with univariate analysis and multivariate regression model. *NLRP3* mutations association with (**E**) tumor heterogeneity, (**F**) purity, and (**G**) ploidy.

In the TCGA cohort, patients with *NLRP3* mutations had significantly higher TMB than patients without (Wilcoxon rank-sum test, *P* < 0.001; Figure 2A). We observed that genome repair regulators including *BRCA1/2*, *TP53*, *POLE*, and MMR genes were frequently mutated and mutations of these genes also induced significantly higher TMB (all *P* < 0.01; Figure 2B). Multivariate Logistic regression model included clinical variables (i.e., age, gender, and stage) and mutations of above genome repair genes was performed to adjust confounding factors. Association of *NLRP3* mutations with higher TMB was still significant (OR: 7.50, 95% CI: 3.85-15.24, *P* < 0.001; Figure 2B). In 2 independent cohorts from ICGC, the Wilcoxon rank-sum test showed that *NLRP3* mutated patients also had significantly higher TMB than *NLRP3* wild-type patients (both *P* < 0.001; Supplementary Figure 1A, 1C). Further multivariate regression model obtained consistent findings after adjusting clinical and genomic confounders (MELA-AU cohort [OR: 8.87, 95% CI: 3.04-29.63, *P* < 0.001]; SKCA-BR cohort [OR: 10.38, 95% CI: 1.38-101.34, *P* = 0.028]; Supplementary Figure 1B, 1D).

Significantly elevated NB was observed in patients with *NLRP3* mutations (Wilcoxon rank-sum test, *P* < 0.001; Figure 2C). Multivariate Logistic model with other confounders taken into account remained statistically significant (OR: 3.94, 95% CI: 1.82-9.04, *P* < 0.001; Figure 2D).

Lower tumor heterogeneity and purity, which suggest the lower proportions of subclonal mutations and tumor cells in microenvironment, were statistically associated with *NLRP3* mutations (Wilcoxon rank-sum test, *P* = 0.048 and *P* = 0.022; Figure 2E, 2F). No significant difference was found in tumor ploidy based on *NLRP3* mutational statuses (Wilcoxon rank-sum test, *P* = 0.49; Figure 2G). The above findings demonstrated that *NLRP3* mutations were linked with favorable genomic characteristics.

### *NLRP3* mutations were correlated with the better microenvironment

**Figure 3:**
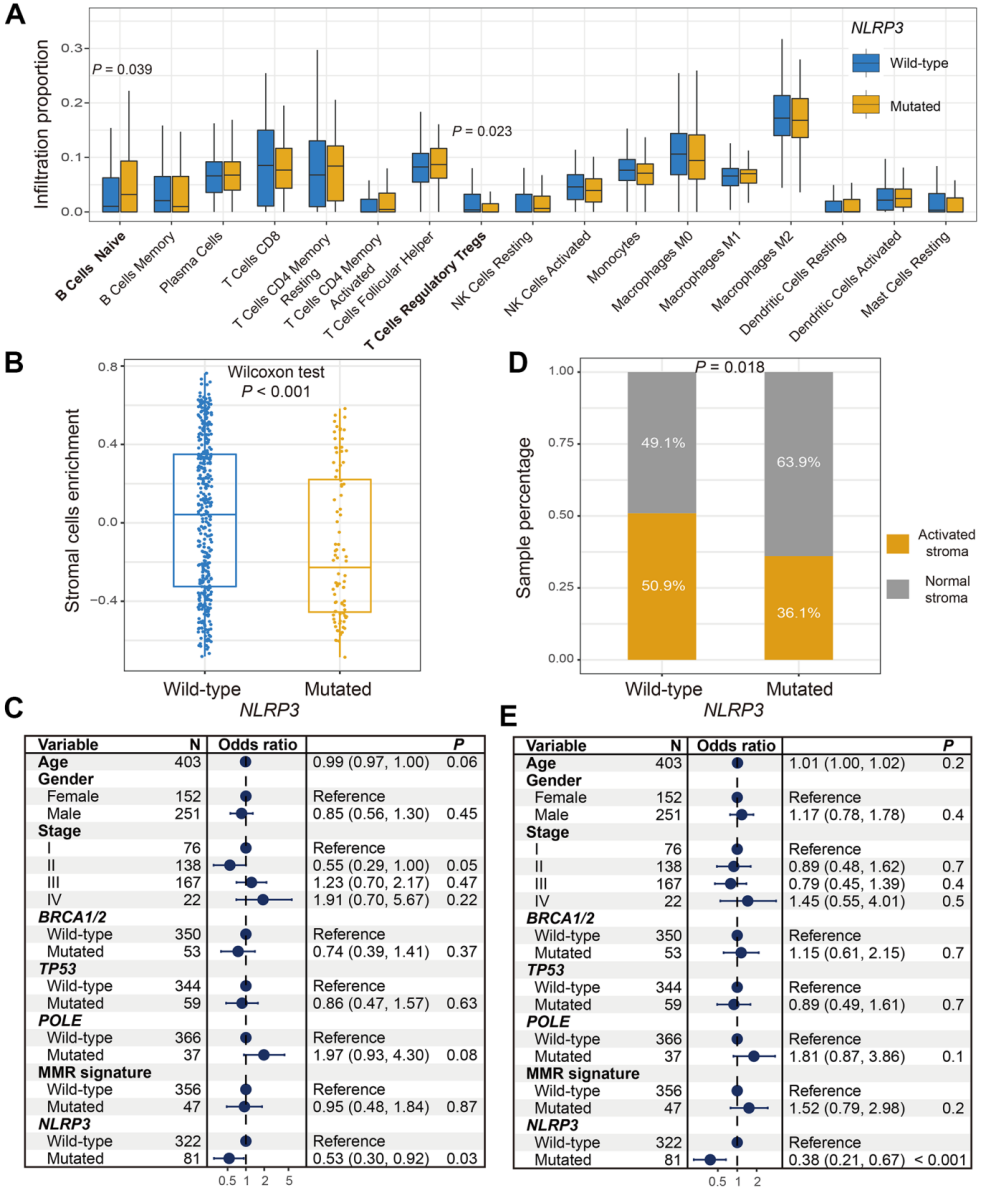
**Association between *NLRP3* mutations and factors in microenvironment.** (**A**) Diverse infiltration abundance of immune cells based on *NLRP3* mutational status. (**B**) Differential enrichment of overall stromal cells in *NLRP3* mutated and wild-type patients. (**C**) Representation for forest plot of association between *NLRP3* mutations and stromal cells enrichment. (**D**) Distinct distribution of activated stroma subtype in patients with and without *NLRP3* mutations. (**E**) Multivariate Logistic regression model for the association of *NLRP3* mutations with activated stroma subtype.

ESTIMATE algorithm showed that the difference of overall immune cells infiltration was not significant between *NLRP3* mutated and wild-type patients (Wilcoxon rank-sum test, *P* = 0.329; Supplementary Figure 2). We thus estimated the abundance of distinct immune cell subtypes using the CIBERSORT approach and compared their differences based on *NLRP3* statuses. Results revealed that significantly lower enrichment of regulatory T cells and higher enrichment of naive B cells were observed in patients with *NLRP3* mutations (Wilcoxon rank-sum test, *P* = 0.023 and *P* = 0.039; Figure 3A). We further calculated the distinct immune cells infiltration according to *NLRP3* mutational statuses via the Angelova et al. method. Consistent with the result of CIBERSORT, significantly lower infiltration of regulatory T cells was also found in patients with *NLRP3* mutations (Wilcoxon rank-sum test, *P* = 0.045; Supplementary Figure 3). In addition, we observed a higher abundance of activated CD4 T cells, effector CD4 T cells, and dendritic cells in *NLRP3* mutant patients (Wilcoxon rank-sum test, all *P* < 0.05; Supplementary Figure 3).

Stromal cells could promote tumor growth and inhibit immune response. Results indicated that patients with *NLRP3* mutations harbored significantly lower enrichment of overall stromal cells (Wilcoxon rank-sum test, *P* < 0.001; Figure 3B). Multivariate Logistic regression model with other confounding factors taken into account still remained significant (OR: 0.53, 95% CI: 0.30-0.92, *P* = 0.03; Figure 3C). Proportion of activated stroma subtypes was significantly decreased in *NLRP3* mutated patients as compared with wild-type patients (proportion: 36.1% vs. 50.9%, Fisher exact test, *P* = 0.018; Figure 3D). This result was more significant in multivariate analysis (OR: 0.38, 95% CI: 0.21-0.67, *P* < 0.001; Figure 3E).

Differential analyses of immune checkpoint genes showed that only *CD276* exhibited a significantly elevated expression in *NLRP3* mutated patients (Wilcoxon rank-sum test, *P* = 0.024; Supplementary Figure 4). Other checkpoint genes did not show statistical significance (Wilcoxon rank-sum test, all *P* > 0.05; Supplementary Figure 4). Collectively, the activated immune microenvironment was enriched in melanoma patients with *NLRP3* mutations.

### Immune response pathways correlated with *NLRP3* mutations

Results of GSEA analysis demonstrated that immune response-related signaling pathways, including graft versus host disease (normalized enrichment score: 2.02, *FDR* = 0.007; Supplementary Figure 5) and allograft rejection (normalized enrichment score: 1.74, *FDR* = 0.024; Supplementary Figure 5) were significantly enriched in the top circuits of *NLRP3* mutations.

### Clinical characteristics versus ICI efficacy in immunotherapy cohort

Before evaluating the association of *NLRP3* mutations with ICI efficacy, we explored the influences of common clinical features (i.e., TMB, age, gender, stage, and treatment type) with 336 ICI-treated melanoma patients. We observed that high TMB was associated with elevated response rate (response rate: 35.7% vs. 25.0%, Fisher exact test, *P* = 0.043) and preferable overall survival (OS) (Log-rank test, *P* = 0.049) (Supplementary Figure 6A). Patients with age > 60 were likely to have a higher response rate than others (response rate: 41.4% vs. 23.7%, Fisher exact test, *P* = 0.002), but they did not exhibit a statistical difference in prognosis (Log-rank test, *P* = 0.942) (Supplementary Figure 6B). Male patients harbored a trend of high response rate, although it did not reach the statistical significance (response rate: 35.9% vs. 25.3%, Fisher exact test, *P* = 0.054); there is no significant difference in OS (Log-rank test, *P* = 0.286) (Supplementary Figure 6C). Patients with advanced-stage had the lowest response rate and worst prognosis, which may be correlated with their intrinsic properties (Supplementary Figure 6D). We found patients treated with anti-PD-1 therapy had the highest response rate than patients treated with anti-CTLA-4 or combined therapy (response rate: 41.6% vs. 31.8% vs. 16.3%, Fisher exact test, *P* = 0.002); nevertheless, patients who received combined therapy had the best OS (Log-rank test, *P* = 0.044) (Supplementary Figure 6E).

### *NLRP3* mutations were linked with favorable ICI efficacy

Associations of *NLRP3* mutations with clinical characteristics among 336 ICI-treated melanoma patients were exhibited in Supplementary Table 1. Consistent with the aforementioned result, significantly increased TMB was identified in patients with *NLRP3* mutations in the immunotherapy cohort (Wilcoxon rank-sum test, *P <* 0.001; Supplementary Figure 7A, 7B).

**Figure 4:**
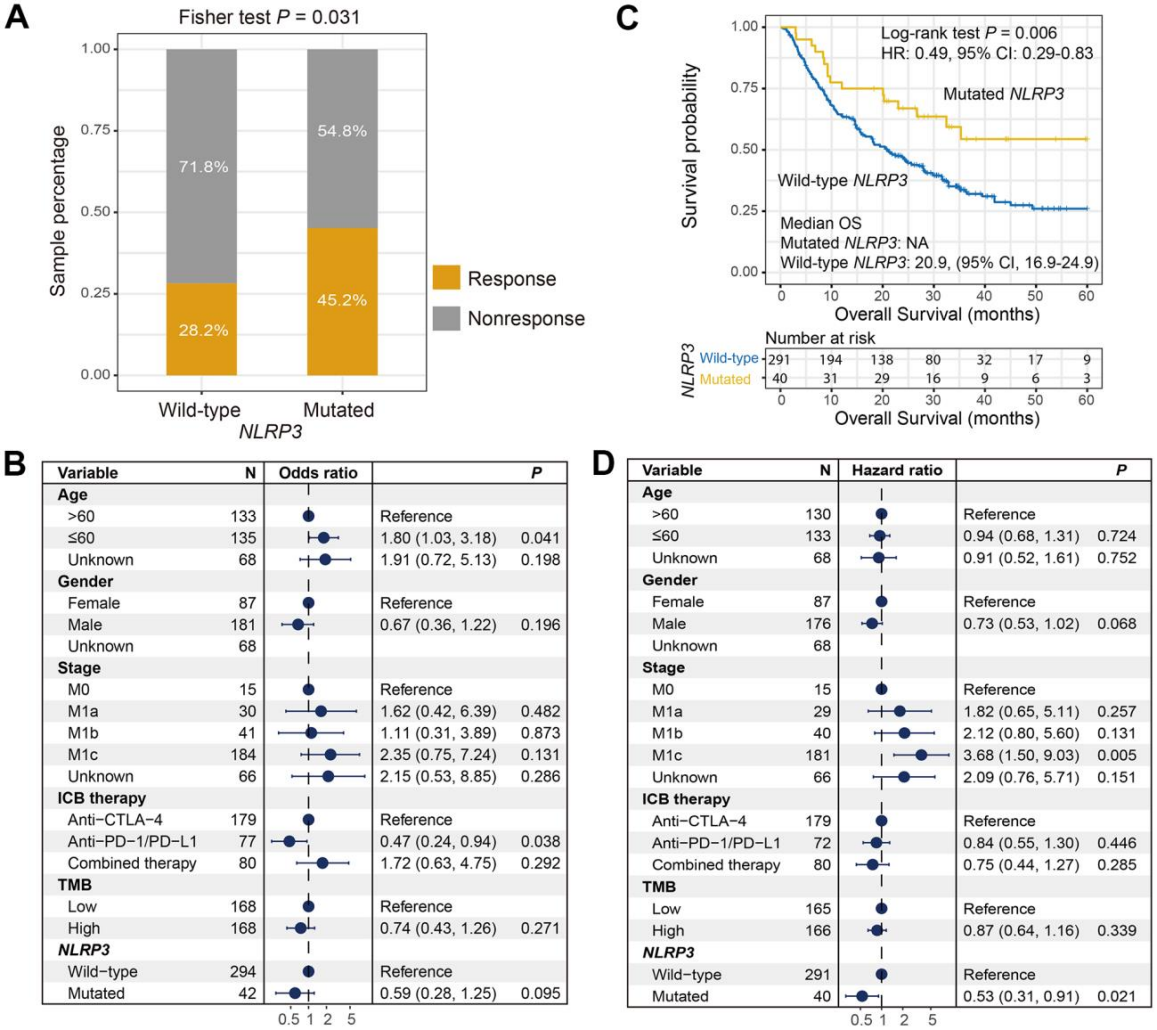
**Correlation of *NLRP3* mutations with ICI response rate and survival interval.** (**A**) Distinct ICI response rate in *NLRP3* mutated and wild-type patients. (**B**) Association of *NLRP3* mutations with the response rate in multivariate Logistic regression model. (**C**) Kaplan-Meier survival curve of distinct *NLRP3* status in ICI-treated cohort. (**D**) Forest plot for multivariate Cox regression model with confounders taken into account.

We found that *NLRP3* mutated patients had significantly higher response rate than wild-type patients (response rate: 45.2% vs. 28.2%, Fisher exact test, *P* = 0.031; Figure 4A). Multivariate Logistic regression model also showed statistical difference after adjusting confounders (i.e., age, gender, stage, ICI therapy type, and TMB) (OR: 0.59, 95% CI: 0.28-1.25, *P* = 0.095; Figure 4B).

We calculated the intrinsic prognostic ability of *NLRP3* mutations using 3 ICI-treated-naive cohorts from TCGA and ICGC. Results showed that *NLRP3* mutations were not correlated with prognoses (Log-rank test, *P* = 0.461, *P* = 0.686 and *P* = 0.916; Supplementary Figure 8). However, survival analysis suggested that *NLRP3* mutated patients had significantly preferable overall survival than wild-type patients in the ICI-treated cohort (median OS: not available [because more than half the patients in this subgroup were alive] vs. 20.9 [95% CI, 16.9-24.9], Log-rank test, *P* = 0.006; Figure 4C). Multivariate Cox regression analysis with clinical variables taken into account still remained significant (HR: 0.53, 95% CI: 0.31-0.91, *P* = 0.021; Figure 4D).

**Figure 5:**
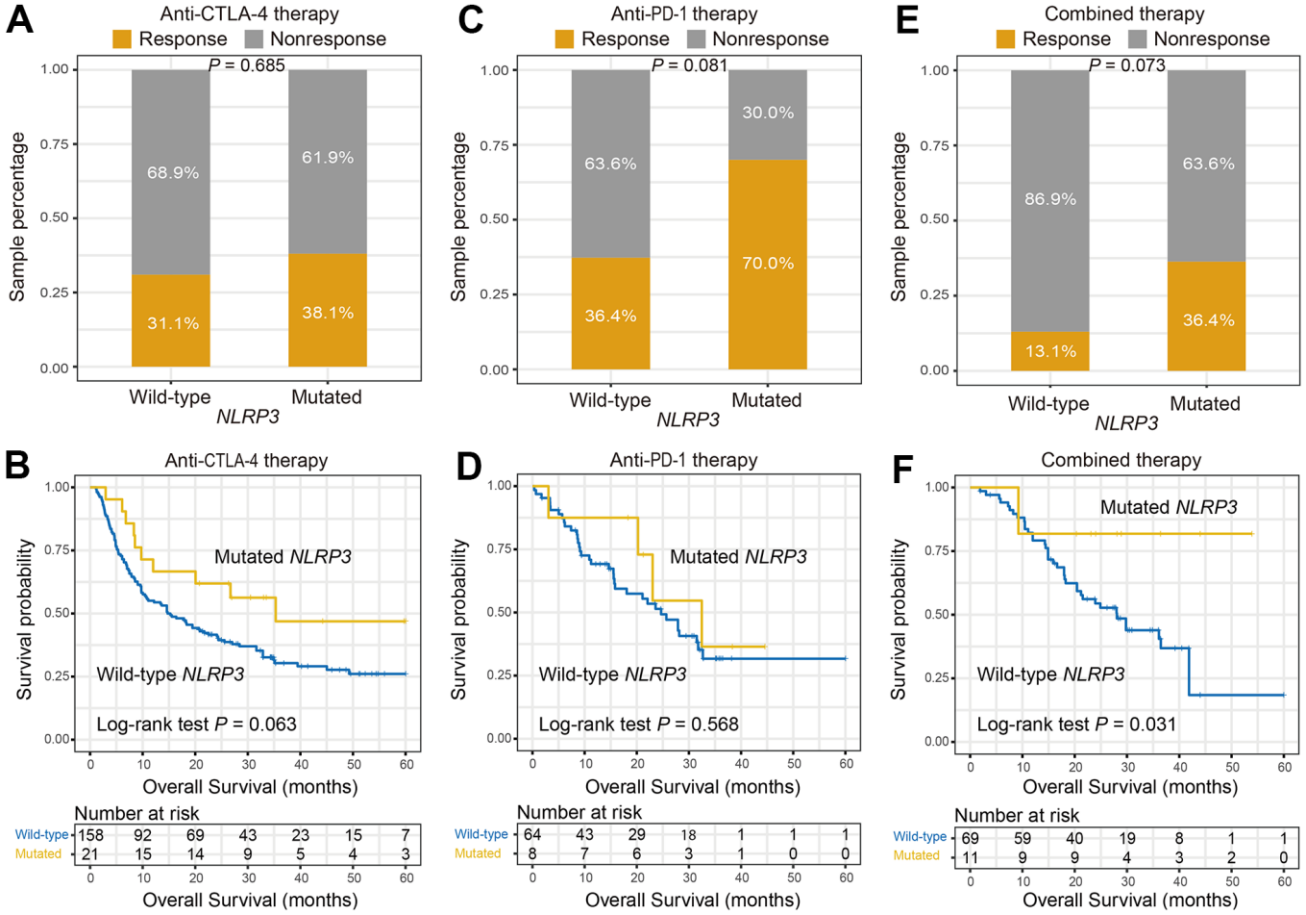
***NLRP3* mutations association with ICI efficacy in distinct therapies.** (**A**, **B**) Association of *NLRP3* mutations with response rate and prognosis in patients treated with anti-CTLA-4 agents. (**C**, **D**) *NLRP3* mutations versus response rate and prognosis in patients treated with anti-PD-1 agents. (**E**, **F**) *NLRP3* mutations versus response rate and prognosis in patients who received combined therapy.

Finally, associations of *NLRP3* mutations with ICI efficacy in distinct therapies were explored respectively. In patients treated with anti-CTLA-4 agents, no association was found between *NLRP3* mutations and response rate (response rate: 38.1% vs. 31.1%, Fisher exact test, *P* = 0.685; Figure 5A); however, *NLRP3* mutations were associated with better OS, although this result did not obtain statistical significance (Log-rank test, *P* = 0.063; Figure 5B). In patients treated with anti-PD-1 agents, we found that patients with *NLRP3* mutations had a marginally significantly higher response rate (response rate: 70.0% vs. 36.4%, Fisher exact test, *P* = 0.081; Figure 5C); correlation of *NLRP3* mutations with OS was not observed (Log-rank test, *P* = 0.568; Figure 5D). In patients received combined therapy, we also found *NLRP3* mutant patients had a marginally significantly elevated response rate (response rate: 36.4% vs. 13.1%, Fisher exact test, *P* = 0.073; Figure 5E); and *NLRP3* mutations were significantly correlated with prolonged immunotherapy OS (Log-rank test, *P* = 0.031; Figure 5F). Collectively, *NLRP3* mutations were predictive of the preferable treatment efficacy in the settings of immunotherapy, especially the combined therapy.

## DISCUSSION

By integrating and analyzing available genomic and clinical data of melanoma, *NLRP3* mutations were identified to be associated with higher mutation and neoantigen burden, favorable microenvironment, and better tumor genomic features. Importantly, our study demonstrated that the elevated response and prolonged survival time of ICI therapy were found in patients with *NLRP3* mutations. These findings suggest the predictive implications of *NLRP3* mutations for melanoma immunotherapy.

Previously many studies revealed the vital roles of mutations of a single gene in evaluating ICI therapy efficacy. Jia et al*.* found that *TTN* mutations were positively associated with ICI predictive biomarkers and immunotherapy survival interval in melanoma and non-small cell lung cancer [26]. Patients with *POLE*/*POLD1* mutations exhibited a significantly preferable prognosis in a multi-cancer-ICI cohort with 1644 patients [27]. In metastatic renal cell carcinoma patients who received Nivolumab antibody, Braun et al*.* observed that favorable overall and progression-free survival were markedly correlated with *PBRM1* mutations [28]. High TMB and NB are 2 promising biomarkers in cancer immune treatment, however, some factors such as uncertain threshold, exome sequencing fees, and bias of different platforms largely influence the precise evaluation for both markers [26]. Mutations of *NLRP3* could accurately assess high TMB and NB as our results described. Therefore, instead of choosing TMB and NB, *NLRP3* mutations may be an alternative surrogate for predicting ICI response in melanoma.

Low tumor heterogeneity and purity suggest the reduced proportion of subclonal mutations and tumor cells in the microenvironment, which were reported to be correlated with better response to anti-PD-1 therapy [29]. Our study found that patients with *NLRP3* mutations had decreased heterogeneity and purity, indicated the vital roles of *NLRP3* mutations in immunotherapy response. The regulatory T cell is one immune cell subtype that performed immune-suppressive roles as well as stromal cells [30, 31]. *NLRP3* mutations were found to be correlated with lower infiltration of regulatory T cells and stromal cells. Moreover, *NLRP3* mutant patients harbored a reduced proportion of activated stroma subtype, which exhibits the roles of immune suppression. The above findings further verify the potentially positive association behind *NLRP3* mutations and ICI therapy response.

In non-ICI-treated cohorts, no survival benefits were found in patients with *NLRP3* mutations. Nevertheless, *NLRP3* mutations exhibited a preferable response rate and ICI efficacy in the aggregated ICI cohort. Noticeably, for the roles of *NLRP3* mutations in specific therapy, we found that *NLRP3* mutated patients could obtain the best survival benefit during combined therapy as compared with anti-CTLA-4 and anti-PD-1 therapies. These findings suggest that *NLRP3* mutations may serve as a predictive indicator for evaluating the efficacy of ICI, especially combined immunotherapy.

Recent research demonstrated that reduced NLRP3 inflammasome and IL-1β expression could inhibit the progression of cancer cells [24]. Consistently, another study reported that NLRP3 downregulation and reduced IL-1β secretion decreased metastatic melanoma by thymoquinone therapy in a mouse model [21]. The above findings suggested the crucial roles of NLRP3 and IL-1β in tumorigenesis and the development of melanoma. In this work, we also evaluated the markedly positive association of *NLRP3* and *IL-1β* expression in melanoma (Supplementary Figure 9A) and further confirmed the collective roles of both regulators. Nevertheless, no significant differences were detected in *NLRP3* wild-type and mutated subgroups with respect to *NLRP3* and *IL-1β* expression (Supplementary Figure 9B, 9C). These results suggested that the mechanisms underlying the association between *NLRP3* mutation and favorable immunotherapy efficacy may neither involve in *NLRP3* nor *IL-1β* transcriptional signals.

A few limitations existed. Firstly, the gene expression-related analyses were performed with only the TCGA cohort, no additionally available datasets were used for validation. Secondly, biological relevance between *NLRP3* mutations and immunological features was elusive, further studies were needed to explore.

In melanoma, *NLRP3* mutations were associated with better immunological and genomic characteristics. It is worth noting that the elevated response rate and favorable ICI survival were also observed in *NLRP3* mutated patients. *NLRP3* mutations may harbor vitally predictive implications for immunotherapy response in melanoma.

## MATERIALS AND METHODS

### Somatic mutation data, gene expression profile, and clinical information of included melanoma patients

Somatic mutation data of 467 melanoma patients in the Cancer Genome Atlas (TCGA) were derived from Genome Data Commons (https://portal.gdc.cancer.gov). MELA-AU and SKCA-BR cohorts respectively containing 183 and 100 patients derived from the International Cancer Genome Consortium (ICGC) (https://dcc.icgc.org) were utilized for specific validation. Gene expression data of 465 patients were acquired from the TCGA cohort.

From previously published 6 studies [1, 32–36], we collected a total of 336 pre-treatment advanced/metastatic melanoma patients with somatic mutation data. These patients were treated with anti-PD-1 agents, anti-CTLA-4 agents, or combined therapy in their trials. All somatic mutations were uniformly annotated with Oncotator [37]. Clinical information (e.g., age, gender, stage, and treatment type) and ICI efficacy (i.e., response status and survival time) of these 336 patients were described in Supplementary Table 2. In this study, patients with statuses of completed or partial response were defined as responders, other statuses including stable and progressive disease were not considered to be efficacious to ICI therapy.

### Association of *NLRP3* mutations with TMB, NB, and genomic features

Mutations of genomic maintenance genes were largely correlated with genome instability. Therefore, in addition to univariate analysis of the association of *NLRP3* mutations with TMB and NB, we also conducted a multivariate Logistic regression model with mutations of DNA damage repair genes (i.e., *TP53*, *BRCA1/2*, and *POLE*) and mismatch repair (MMR) genes (i.e., *MLH1*, *MSH2*, *MSH6*, and *PMS2*) taken into account to control false positive. The neoantigen data of 340 patients were acquired from the Cancer Immunome Atlas (TCIA, https://www.tcia.at/home). TMB and NB were stratified into high and low subgroups with the median.

A number of studies have reported the vital roles of tumor genomic features (i.e., heterogeneity, purity, and ploidy) on immune response and immunotherapeutic efficacy [29, 38]. We therefore utilized relevant data of 140 patients derived from TCIA to evaluate the association of *NLRP3* mutations with heterogeneity and ploidy. For the tumor purity of each patient, we used the ESTIMATE algorithm embedded in R package ESTIMATE [39] to calculate.

### *NLRP3* mutations versus tumor microenvironment

Overall infiltration of immune and stromal cells of each sample was evaluated with the aforementioned ESTIMATE algorithm. The nearest template prediction (NTP) algorithm [40] with a 48-gene signature [41] was applied to stratify melanoma patients into activated and normal stroma subgroups. A recent study reported that the activated stroma subtype exhibited an immune-suppressive role and a worse prognosis [41].

The infiltration abundance of 17 immune cell types was estimated with the CIBERSORT algorithm [42]. Angelova et al. established 812 immune metagene signatures to infer 31 immune cells infiltration and tumor immune landscape [43]. We used both methods to obtain comprehensive results and to validate each other.

An integrated list of 33 immune checkpoint genes was acquired from a recently published study [44]. We analyzed the distinct distributions of the above immune cells and checkpoint genes based on *NLRP3* mutational statuses. All analyses in this section were performed with gene expression data of 465 samples from TCGA.

### GSVA and GSEA

Single sample gene set enrichment analysis (ssGSEA) function embedded in GSVA package (V1.36.1) [45] was utilized to calculate the enrichment of a specific gene set for each patient. Differential analysis according to *NLRP3* mutational statuses was performed with R package DESeq2 (V1.28.1) [46], which manages sequencing expression data. The *t* values obtained from differential analysis were subsequently used to conducted gene set enrichment analysis (GSEA) implemented by fgsea package (V1.14.0) (https://github.com/ctlab/fgsea). Kyoto encyclopedia of genes and genomes (KEGG) pathways were utilized as the background dataset.

### Statistical analyses

R software (V4.0.1) and its affiliated packages were downloaded to complete related calculations and analyses. Waterfall plot of mutational patterns was achieved through GenVisR package (V1.20.0) [47]. Kaplan-Meier survival curve was drawn with survival (V2.41-3) and survminer (V0.4.7) packages and Log-rank test to compare the difference. We used forestmodel package (V0.5.0) to perform multivariate regression analyses and to produce forest plots. Correlations of *NLRP3* mutations with continuous and categorical factors were calculated with Wilcoxon rank-sum test and Fisher exact test, separately.

## Supplementary Material

Supplementary Figures

Supplementary Table 1

Supplementary Table 2
