# Supplementary material for: Immunological and clinical immunotherapy implications of NLRP3 mutations in melanoma
Source: Aging (Albany NY). 2021 Nov 8;13(21):24271–89. doi: 10.18632/aging.203678 (PMC8610131; doi:10.18632/aging.203678)
Supplement: Supplementary Figures [file aging-13-203678-s001.pdf]

SUPPLEMENTARY FIGURES

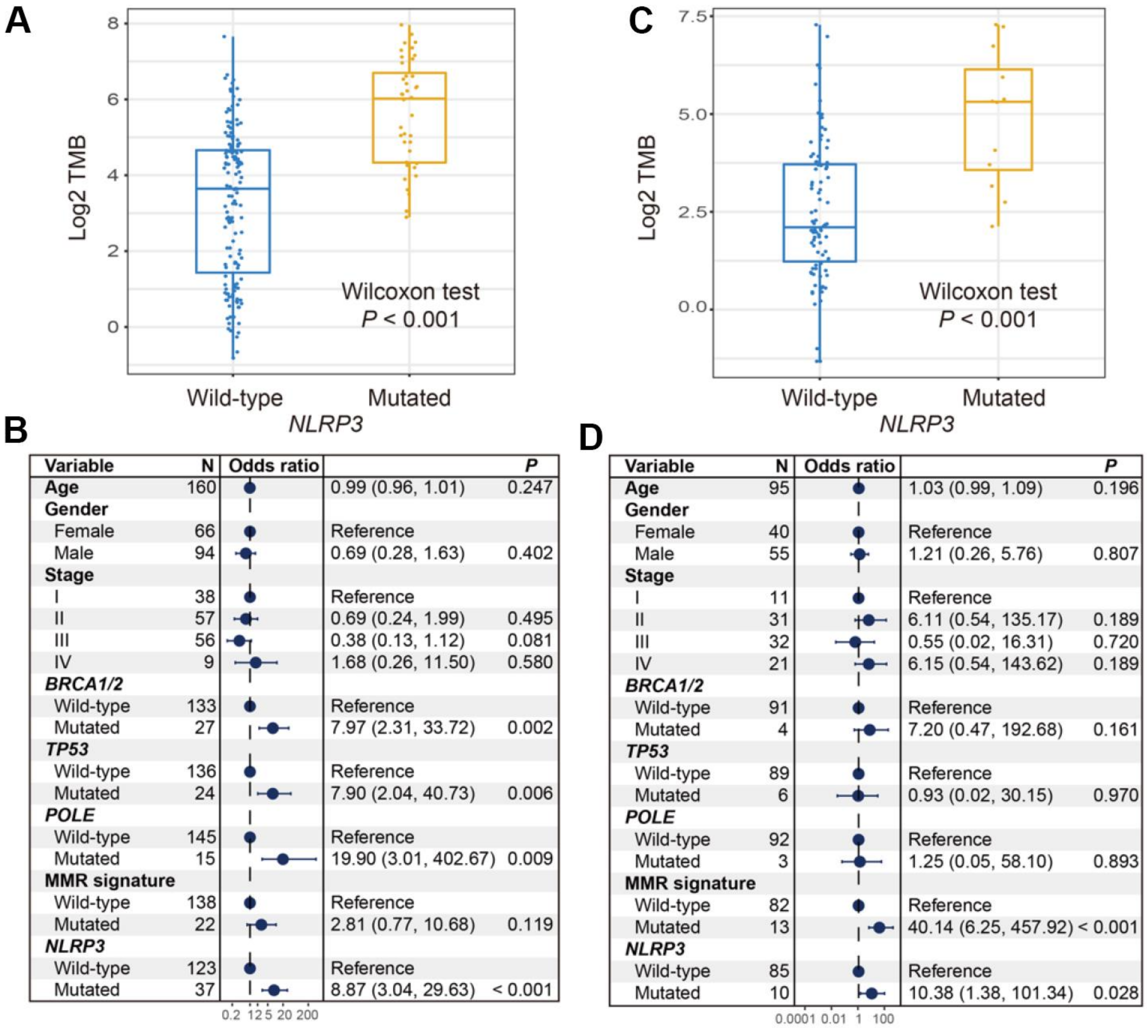

Supplementary Figure 1. Association of *NLRP3* mutations with TMB in 2 ICGC melanoma cohorts. *NLRP3* mutations versus TMB with (**A**) univariate analysis and (**B**) multivariate regression model in MELA-AU cohort; *NLRP3* mutations versus TMB with (**C**) univariate analysis and (**D**) multivariate regression model in SKCA-BR cohort.

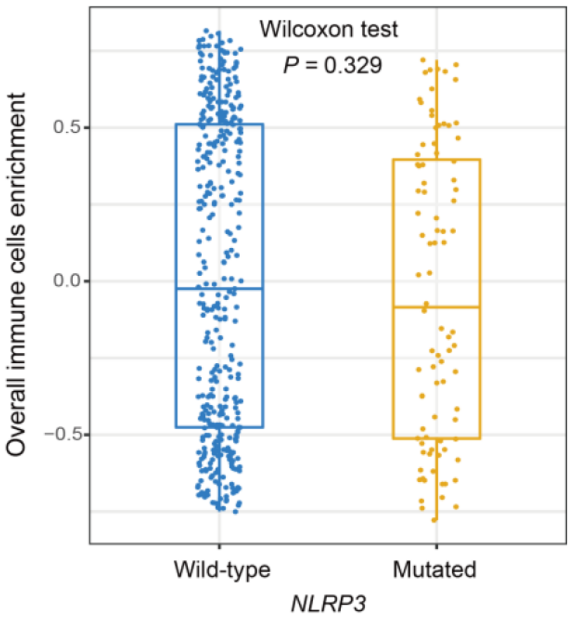

Supplementary Figure 2. Infiltration of overall immune cells in patients with and without *NLRP3* mutations.

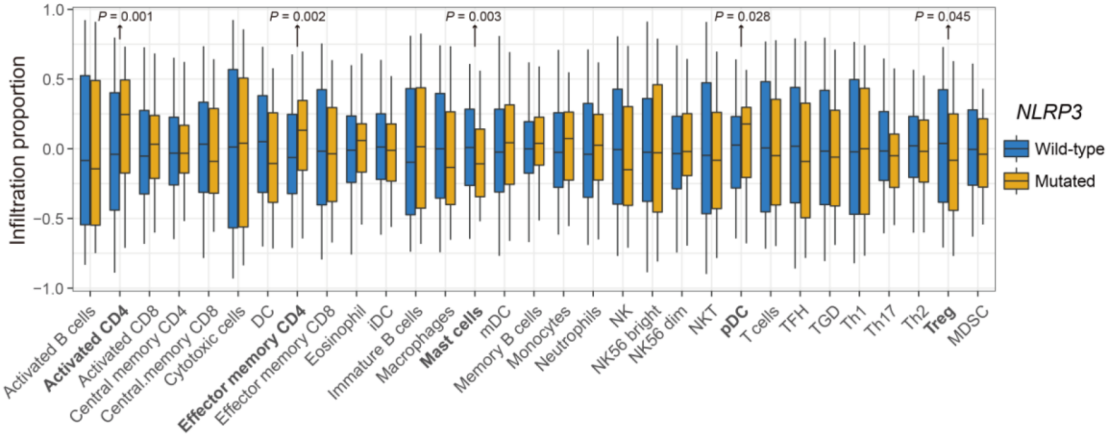

Supplementary Figure 3. Infiltration of 31 immune cells stratified by *NLRP3* mutation status according to Angelova et al. method.

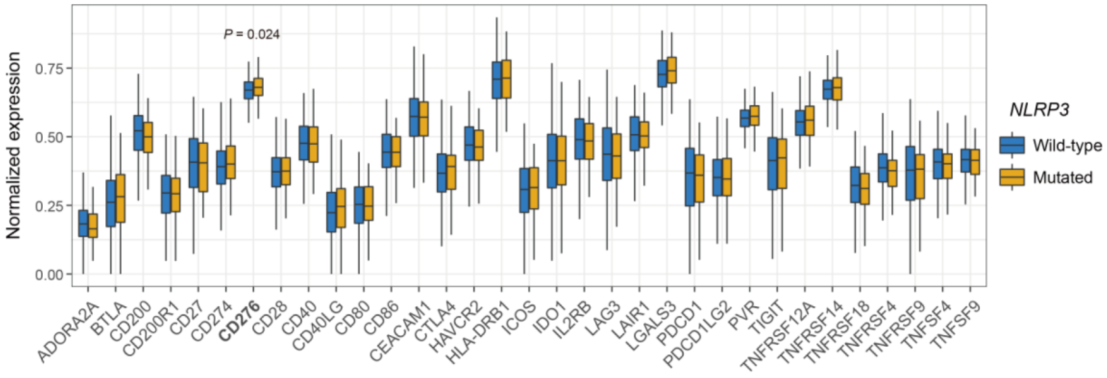

Supplementary Figure 4. Expression of 33 immune checkpoint genes stratified by *NLRP3* mutation status.

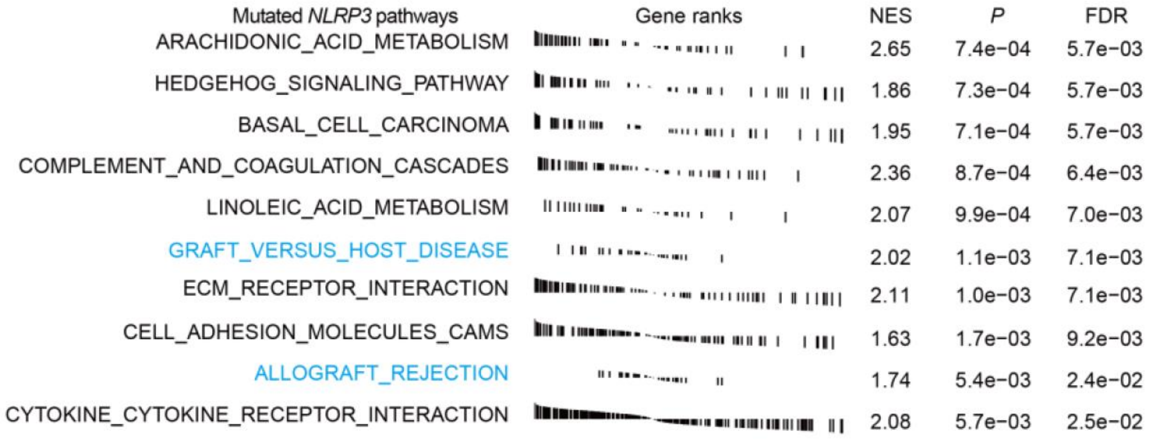

Supplementary Figure 5. GSEA analysis of *NLRP3* mutant patients.

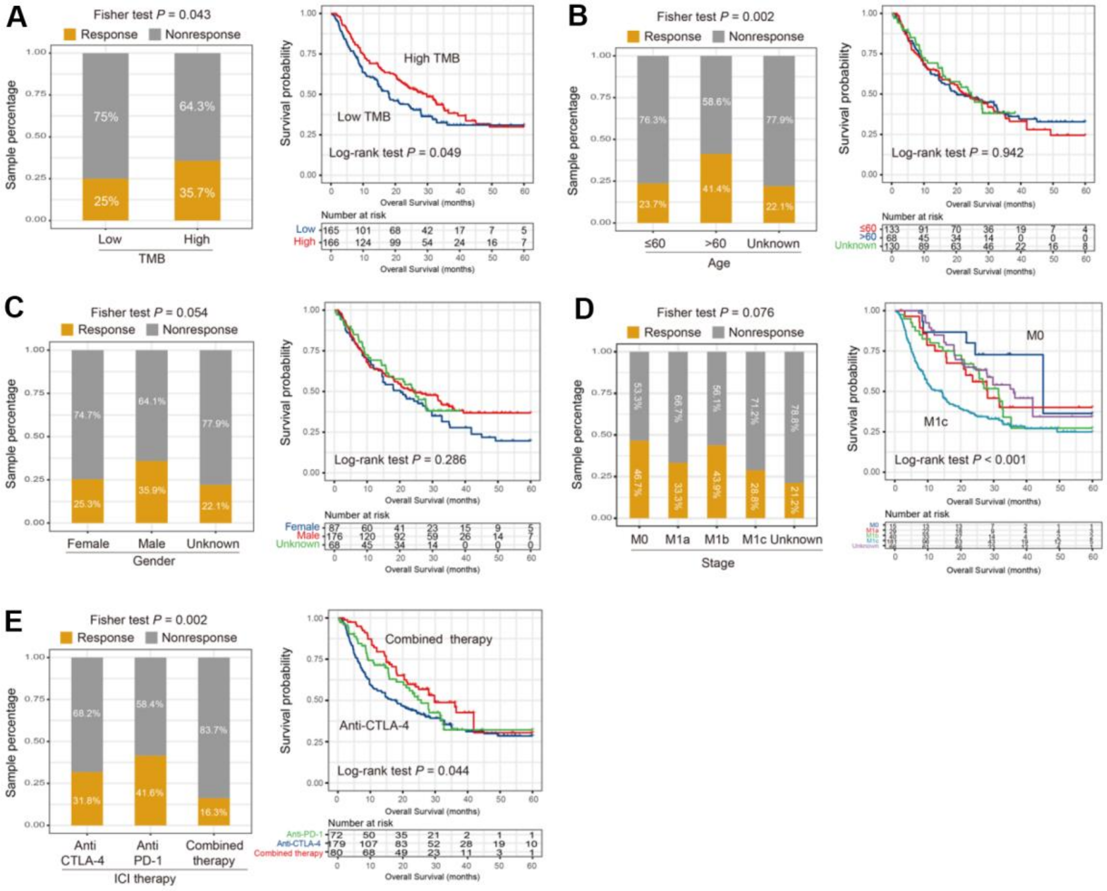

Supplementary Figure 6. Clinical characteristics association with ICI efficacy. Association of (**A**) TMB, (**B**) age, (**C**) gender, (**D**) stage, and (**E**) treatment type with ICI response rate and prognosis.

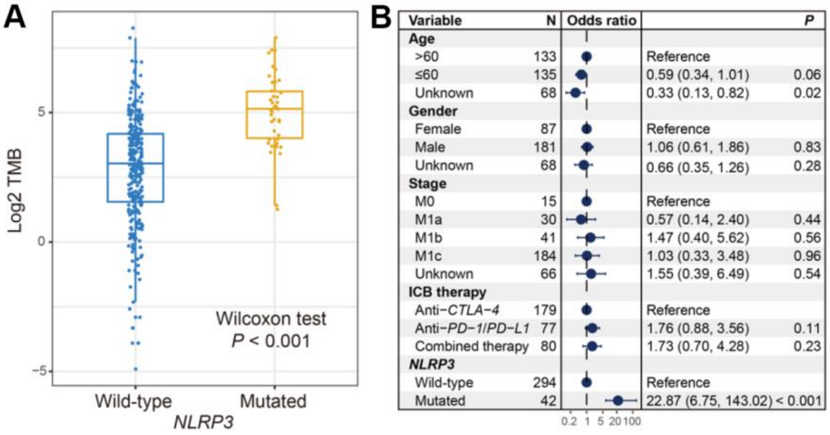

Supplementary Figure 7. Correlation of *NLRP3* mutations with TMB in the pooled ICI-treated cohort. (**A**) Wilcoxon rank-sum test and (**B**) multivariate Logistic regression analysis for the correlation of *NLRP3* mutations with TMB.

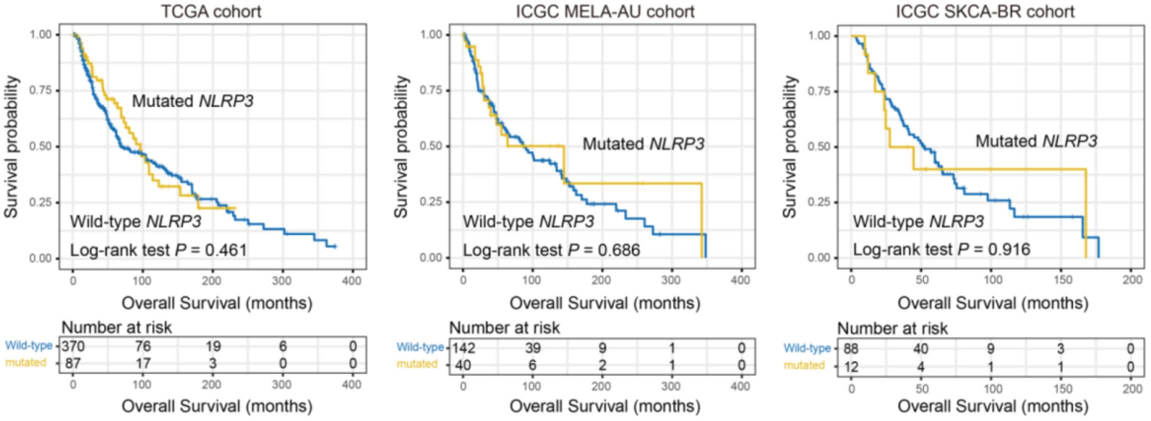

Supplementary Figure 8. Kaplan-Meier survival analysis of *NLRP3* mutations in 3 non-ICI-treated cohorts.

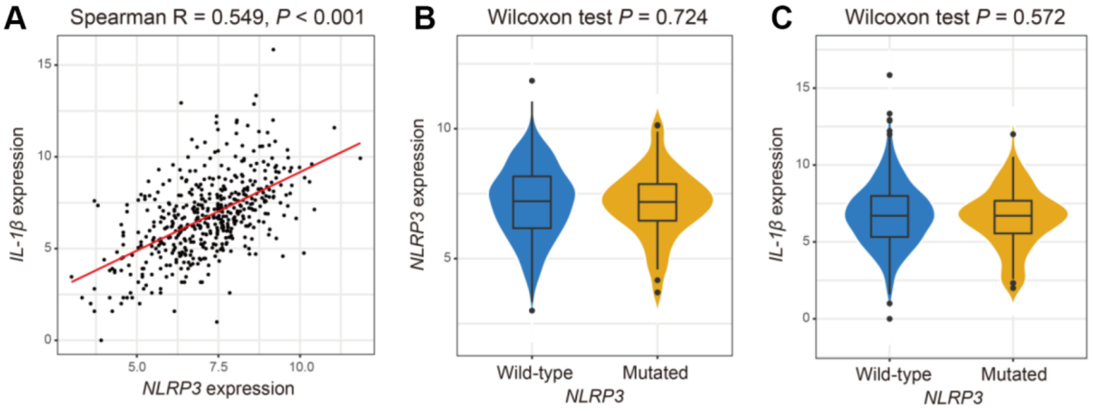

Supplementary Figure 9. *NLRP3* and *IL-1β* mRNA expression concerning *NLRP3* mutational status in melanoma based on the data from TCGA cohort. (**A**) Correlation analysis between *NLRP3* and *IL-1β* expression. Distinct expression of (**B**) *NLRP3* and (**C**) *IL-1β* in *NLRP3* wild-type versus mutated subgroups.
