# Supplementary material for: Immunological and clinical immunotherapy implications of NLRP3 mutations in melanoma
Source: Aging (Albany NY). 2021 Nov 8;13(21):24271–89. doi: 10.18632/aging.203678 (PMC8610131; doi:10.18632/aging.203678)
Supplement: Supplementary Table 1 [file aging-13-203678-s002.pdf]

SUPPLEMENTARY TABLE

Supplementary Table 1. Association of *NLRP3* mutations with clinical characteristics in 336 ICI-treated melanoma patients.

Characteristics	n (%) (N = 366)	*NLRP3*		*P*
		Wild-type (n = 294)	Mutated (n = 42)	
Age, years				
≤60	135 (40.2%)	122 (41.5%)	13 (30.9%)	
>60	133 (39.6%)	110 (37.4%)	23 (54.8%)	0.112
Unknown	68 (20.2%)	62 (21.1%)	6 (14.3%)	
Gender				
Male	181 (53.9%)	152 (51.7%)	29 (69.0%)	
Female	87 (25.9%)	80 (27.2%)	7 (16.7%)	0.119
Unknown	68 (20.2%)	62 (21.1%)	6 (14.3%)	
Stage				
M0	15 (4.5%)	13 (4.4%)	2 (4.8%)	
M1a	30 (8.9%)	29 (9.9%)	1 (2.4%)	
M1b	41 (12.2%)	36 (12.2%)	5 (11.8%)	0.343
M1c	184 (54.8%)	162 (55.1%)	22 (52.4%)	
Unknown	66 (19.6%)	54 (18.4%)	12 (28.6%)	
ICB treatment				
Anti-CTLA-4	179 (53.3%)	158 (53.7%)	21 (50.0%)	
Anti-PD-1	77 (22.9%)	67 (22.8%)	10 (23.8%)	0.882
Combined therapy	80 (23.8%)	69 (23.5%)	11 (26.2%)	
RECIST				
CR/PR	102 (30.4%)	83 (28.2%)	19 (45.2%)	
SD	43 (12.8%)	39 (13.3%)	4 (9.5%)	0.229
PD	191 (56.8%)	172 (58.5%)	19 (45.3%)	
OS status				
Alive	132 (39.3%)	108 (36.7%)	24 (57.1%)	
Dead	200 (59.5%)	184 (62.6%)	16 (38.1%)	0.002
Unknown	4 (1.2%)	2 (0.7%)	2 (4.8%)	
TMB				
Low	168 (50.0%)	166 (56.5%)	2 (4.8%)	< 0.001
High	168 (50.0%)	128 (43.5)	40 (95.2%)	
